# Evaluation of the anterior chamber angle by anterior segment optical coherence tomography after implantable phakic contact lens implantation in myopic eyes

**DOI:** 10.1007/s10792-022-02244-2

**Published:** 2022-03-04

**Authors:** Mohamed Salah El-Din Mahmoud, Asmaa Anwar Mohamed, Hosny Ahmed Zein

**Affiliations:** Ophthalmology Department, Faculty of Medicine, Minia University, Minia, Egypt

**Keywords:** Anterior chamber (AC) angle, Anterior segment optical coherence tomography (AS-OCT), Implantable phakic contact lens (IPCL)

## Abstract

**Purpose:**

To evaluate the changes in the angle of the AC and lens vault after IPCL implantation by AS-OCT in myopic patients.

**Methods:**

This was a prospective observational study involving 30 myopic eyes implanted with IPCL. AS-OCT was used to evaluate lens vault and AC angle parameters including anterior chamber angle, angle opening distance and trabecular-iris space area (TISA) at 1, 3 and 6 months postoperatively.

**Results:**

All 3 AC angle parameters were significantly reduced at the 1st postoperative month compared to preoperative values, but remained stable thereafter with no significant change at the 3rd or 6th postoperative months. The lens vault showed no significant change over the entire follow-up period.

**Conclusion:**

IPCL implantation is a safe method for correction of myopia with stable AC angle narrowing over the course of 6 months postoperatively as monitored using AS-OCT.

## Introduction

Phakic posterior chamber intraocular lenses (pIOLs) can be used for correction of refractive errors in patients seeking refractive surgery who are deemed unfit for corneal refractive procedures such as those having high errors and/or thin corneas. These lenses have the added advantages of better postoperative contrast sensitivity and lower induced postoperative aberrations. Nevertheless, they carry the risks of intraocular surgery including potential trauma to intraocular structures, retinal detachment and endophthalmitis [1, 2].

Currently, the most commonly implanted pIOLs are the Visian Implantable Collamer Lens (ICL-Staar Surgical AG, Nidau, Switzerland) and the Implantable Phakic Contact Lens (IPCL-Caregroup Sight Solutions, India). The ICL is about 2.5 more expensive than the IPCL which can pose an economic burden for patients in developing countries [3, 4]. The IPCL is a foldable posterior chamber IOL designed to be implanted in the ciliary sulcus. It requires a 2.8-mm corneal incision for implantation. The currently available design is V2.0 which has a central hole of 350 µm that obviates the need for a peripheral iridectomy (PI), unlike the former design (V1) that required a PI to ensure proper unobstructed aqueous circulation [5].

Optical coherence tomography (OCT) was first used to image the retina and optic nerve head, but it has been rapidly adapted to image the anterior segment and cornea [6]. Our study aims to evaluate the anterior chamber iridocorneal angle parameters and lens vault before and after IPCL implantation using anterior segment OCT (AS-OCT).

## Materials and methods

### Study population

This was a prospective interventional case series done in the Department of Ophthalmology, Minia University Hospital, Minia, Egypt, in the period from April 2019 to December 2020. The study protocol was approved by the local Ethical Committee of the Faculty of Medicine, Minia University, and a written informed consent was obtained from all study participants after thorough explanation of the nature of the study and potential benefits and risks of the surgical procedure. Patients 18 years of age or older were included if they had ≥ − 8 D of myopia and ≤ − 2 D of astigmatism. Patients had to have a stable refraction over the course of the year prior to inclusion and an anterior chamber depth (ACD) of ≥ 3 mm to be included in the study. Patients were excluded if they had a corneal endothelial count < 2500 cells/mm^2^, an intraocular pressure (IOP) > 21 mmHg, history of prior intraocular surgery, any coexisting corneal, retinal or optic nerve pathology, diabetes mellitus (DM) or autoimmune disease.

### Preoperative evaluation

All patients underwent a comprehensive ophthalmologic evaluation including slit-lamp examination of the anterior segment, automated refraction, measurement of Snellen uncorrected (UCDVA) and best-corrected distance visual acuity (BCDVA), IOP measurement using Goldmann applanation tonometry (GAT) and fundus examination using both slit-lamp biomicroscopy with a non-contact + 90 D lens and binocular indirect ophthalmoscopy (BIO).

All eyes had an endothelial cell count using NIDEK CEM-530 specular microscope (NIDEK CO., LTD., Aichi, Japan) and Scheimpflug corneal tomography using Oculus Pentacam HR (Oculus; Optikgeräte GmbH, Wetzlar, Germany). Refraction as well as Pentacam-measured keratometry readings, corneal pachymetry, internal anterior chamber depth (ACD) and white-to-white diameter (WTW) were entered into the IPCL online manufacturer’s software to calculate the ideal IPCL power and size.

### Surgical technique

All surgeries were performed by a single experienced surgeon under general anesthesia (GA) as study subjects were relatively young age and anxious. Preoperative pupillary dilatation was achieved using tropicamide 1% and phenylephrine hydrochloride 2.5%. Loading of the IPCL V2.0 was done before making the corneal incision. The IPCL was implanted via a 2.8-mm temporal clear corneal incision after injection of viscoelastic. After implantation, the footplates were tucked under the iris using a spatula, followed by injection of an intracameral miotic and then washout of the viscoelastic. Finally, wound hydration was performed.

Postoperative treatment consisted of topical moxifloxacin hydrochloride 0.5% (Vigamox, Novartis, Basel, Switzerland) and topical prednisolone acetate 1% (Orchapred suspension, Orchidia Pharmaceutical, Cairo, Egypt) in tapering doses. Topical antiglaucoma eye drops as beta-blockers were used in some cases.

### AS-OCT imaging

Anterior segment imaging was done using the Avanti RTVue-XR platform (Optovue, Fremont, CA, USA) spectral domain OCT with the add-on lens of the corneal adaptor module (CAM-L mode: S/N 43386).

AS-OCT imaging was performed in dim illumination. The enhanced anterior segment single protocol was used. The video image was centered on the limbus in 4 quadrants (nasal, temporal, superior, inferior), and the scan head was moved toward the patient until the anterior chamber angle view came to focus. The patient was asked to look into the imaging aperture and at the center of the blue star-shaped target, and then images were captured by pressing the joystick or checkmark button. The scleral spur was identified as inward projection of the sclera as the junction between the inner scleral and corneal curvature. Three measurements were taken from each image and their average recorded. The following AC angle parameters were measured:

- *Anterior chamber angle (ACA)* The trabecular-iris angle estimated at 750 μm from the scleral spur with the apex in the iris recess and the angle arms passing through a point on the trabecular meshwork and the point on the iris perpendicularly opposite, measured in degrees of arc.
- *Angle opening distance at 750 µm (AOD750)* The distance between the posterior corneal surface and the anterior iris surface on a line perpendicular to the trabecular meshwork, measured in microns at 750 μm from the scleral spur.
- *Trabecular-iris space area at 750 μm (TISA750)* A trapezoid surface area with the following boundaries: anteriorly, the opening angle 750 μm away from the scleral spur; posteriorly, the line traced from the scleral spur perpendicular to the iris plane of the inner scleral wall; superiorly, the inner corneoscleral wall; and inferior, the surface of the iris, measured in squared millimeters.

The vault in microns was manually measured by drawing a line from the middle of the IPCL’s back surface and the crystalline lens's anterior surface.

### Statistical analyses

All statistical analyses were done using IBM SPSS Statistics for Windows, version 25.0 (IBM Corp., Armonk, NY, USA). Snellen visual acuity measurements were converted into LogMAR for statistical analyses. Mean ± standard deviation (range) was used to describe parametric quantitative data, while number (percentage) was used to describe categorical data. For comparison of dependent quantitative data, the dependent (paired) sample *t* test was used. Statistical significance was accepted at *P* < 0.05.

## Results

### Demographic data

We included 30 myopic eyes of 30 patients, 16 males and 14 females that were implanted with IPCL ranging from 22 to 32 years of age.

### Visual outcome

The preoperative UCDVA was 0.04 ± 0.01 which improved to 0.34 ± 0.09 after 6 months follow-up. The BCDVA was 0.36 ± 0.09 which improved to 0.32 ± 0.07 after 6 months follow-up. The visual and refractive outcomes are summarized in Table 1.

**Table 1:** Visual and refractive outcome

Variable	Preoperative	First follow-up (1 month)	Second follow-up (3 months)	Third follow-up (6 months)	*P*-value
UCDVA (Log MAR)	1.4 ± 2	0.51 ± 1.1	0.46 ± 0.96	0.47 ± 1.05	*P*1: < 0.001 *P*2: < 0.001 *P*3: < 0.001 *P*4: 0.003 *P*5: 0.010 *P*6: 0.161
Sphere (Diopter)	−12.5 ± 2.8	− 0.70 ± 0.24	− 00.53 ± 0.08	− 0.50 ± 0.14	*P*1: < 0.001 *P*2: < 0.001 *P*3: < 0.001 *P*4: 0.001 *P*5: 0.008 *P*6: 0.425
Cylinder (Diopter)	− 0.82 ± 0.54	− 0.48 ± 0.20	− 0.44 ± 0.19	− 0.51 ± 0.23	*P*1: 0.016 *P*2: 0.004 *P*3: 0.015 *P*4: 0.381 *P*5: 0.537 *P*6: 0.275
BCDVA	0.44 ± 1.05	0.48 ± 1.1	0.47 ± 1.05	0.49 ± 1.15	*P*1: 0.048 *P*2: 0.236 *P*3: 0.058 *P*4: 0.653 *P*5: 0.442 *P*6: < 0.001

*UCDVA* Uncorrected distant visual acuity, *BCDVA* best-corrected distant visual acuity

### Intraocular pressure (IOP)

IOP increased significantly in the first follow-up (1 month) and returned to near the preoperative value in the second and third follow-up visits (Table 2).

**Table 2:** IOP changes

	Preoperative	First follow-up (1 month)	Second follow-up (3 month)	Third follow-up (6 months)	
IOP (mmHg)	13.47 ± 1.27	18.40 ± 1.22	15.2 ± 1.71	13.33 ± 1.76	*P*1: < 0.001 *P*2: < 0.001 *P*3: 0.696 *P*4: < 0.001 *P*5: < 0.001 *P*6: < 0.001

*P*1, pre versus first; *P*2, pre versus second; *P*3, pre versus third; *P*4, first versus second; *P*5, first versus third; *P*6, second versus third

### AC angle parameters

#### Anterior chamber angle (ACA)

**Fig. 1:**
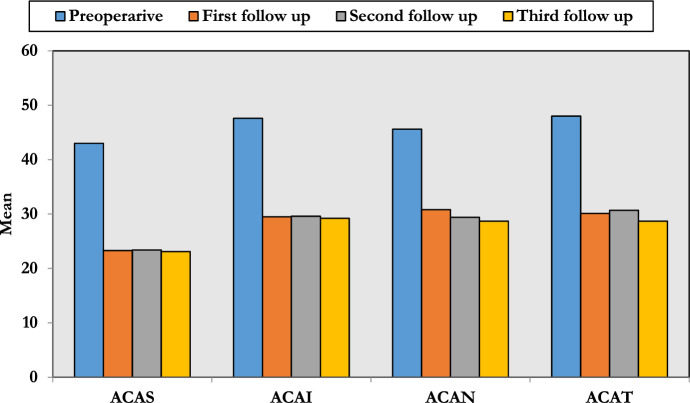
Changes in the ACA

The preoperative values for superior (ACAS), inferior (ACAI), nasal (ACAN) and temporal (ACAT) quadrants were 43 ± 1.28, 47.6 ± 3.92, 45.6 ± 1.38 and 48.0 ± 2.11 degrees, respectively, and decreased to 23.3 ± 3.3, 29.5 ± 5.09, 30.8 ± 3.7 and 30.1 ± 5.3 degrees, respectively, after 1 month which was statistically significant with no significant change after 3 or 6 months (Table 3 and Fig. 1).

**Table 3:** Anterior chamber angle changes (ACA)

Variable	Preoperative	First follow-up (1 month)	Second follow-up (3 month)	Third follow-up (6 months)	*P*-value
ACAS (degree)	43 ± 1.28	23.3 ± 3.3	23.4 ± 2.9	23.1 ± 3.5	*P*1 : < 0.001 *P*2: < 0.001 *P*3: < 0.001 *P*4: 0.671 *P*5: 0.438 *P*6: 0.326
ACAI (degree)	47.6 ± 3.92	29.5 ± 5.09	29.6 ± 2.4	29.2 ± 2.7	*P*1: < 0.001 *P*2: < 0.001 *P*3: < 0.001 *P*4: 0.890 *P*5: 0.781 *P*6: 0.062
ACAN (degree)	45.6 ± 1.38	30.8 ± 3.7	29.4 ± 4.1	28.7 ± 3.9	*P*1: < 0.001 *P*2: < 0.001 *P*3: < 0.001 *P*4: 0.071 *P*5: 0.058 *P*6: 0.066
ACAT (degree)	48.0 ± 2.11	30.1 ± 5.3	30.7 ± 4.1	28.7 ± 3.9	*P*1: < 0.001 *P*2: < 0.001 *P*3: < 0.001 *P*4: 0.070 *P*5: 0.537 *P*6: 0.120

*P*1, pre versus first; *P*2, pre versus second; *P*3, pre versus third; *P*4, first versus second; *P*5, first versus third; *P*6, second versus third

*ACAS* anterior chamber angle superior, *ACAI* anterior chamber angle inferior, *ACAN* anterior chamber angle nasal, *ACAT* anterior chamber angle temporal

#### Angle opening distance (AOD)

**Fig. 2:**
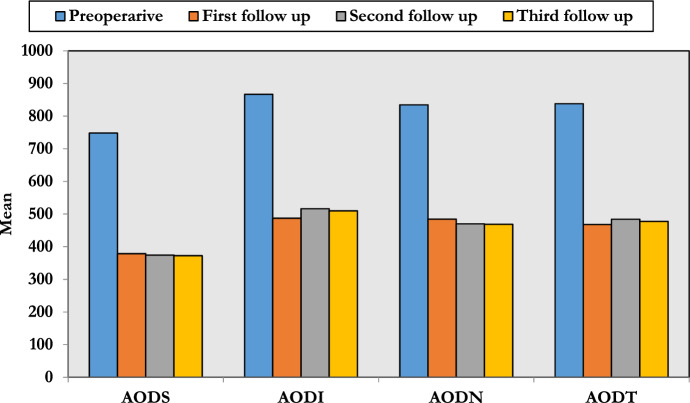
Changes in the AOD

The preoperative values for superior (AODS), inferior (AODI), nasal (AODN) and temporal (AODT) quadrants were 748.2 ± 41.7, 866.7 ± 48.2, 834.4 ± 23.9 and 837.7 ± 37.2 μm, respectively, and decreased to 378.6 ± 56.3, 487.2 ± 82.9, 484.4 ± 75.9 and 467.9 ± 80.4 μm, respectively, after 1 month which was statistically significant with no significant change after 3 or 6 months (Table 4 and Fig. 2).

**Table 4:** Angle opening distance changes AOD

Variable	Preoperative	First follow-up	Second follow-up	Third follow-up (6 months)	*P*-value
AODS (μm)	748.2 ± 41.7	378.6 ± 56.3	374.5 ± 57.1	372.3 ± 64.9	*P*1: < 0.001 *P*2: < 0.001 *P*3: < 0.001 *P*4: 0.072 *P*5: 0.067 *P*6: 0.323
AODI (μm)	866.7 ± 48.2	487.2 ± 82.9	516.3 ± 46.6	509.8 ± 47.8	*P*1: < 0.001 *P*2: < 0.001 *P*3: < 0.001 *P*4: 0.061 *P*5: 0.054 *P*6: 0.062
AODN (μm)	834.4 ± 23.9	484.4 ± 75.9	470 ± 77.8	468.5 ± 78.0	*P*1: < 0.001 *P*2: < 0.001 *P*3: < 0.001 *P*4: 0.077 *P*5: 0.121 *P*6: 0.090
AODT (μm)	837.7 ± 37.2	467.9 ± 80.4	484.1 ± 59.4	477.7 ± 62.2	*P*1: < 0.001 *P*2: < 0.001 *P*3: < 0.001 *P*4: 0.065 *P*5: 0.175 *P*6: 0.081

*P*1, pre versus first; *P*2, pre versus second; *P*3, pre versus third; *P*4, first versus second; *P*5, first versus third; *P*6, second versus third

*AODS* angle opening distance superior, *AODI* angle opening distance inferior, *AODN* angle opening distance nasal, *AODT* angle opening distance temporal

#### Trabecular-iris space area at 750 μm (TISA)

**Fig. 3:**
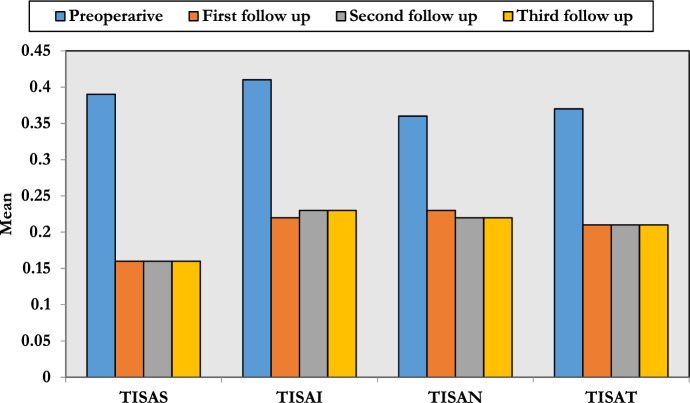
Changes in the TISA

The preoperative values for superior (TISAS), inferior (TISAI), nasal (TISAN) and temporal (TISAT) quadrants were 0.39 ± 0.04, 0.41 ± 0.04, 0.36 ± 0.01 and 0.37 ± 0.03 mm^2^, respectively, and decreased to 0.16 ± 0.03, 0.22 ± 0.04, 0.23 ± 0.06 and 0.21 ± 0.06 mm^2^, respectively, after 1 month which was statistically significant with no significant change after 3 or 6 months (Table 5 and Fig. 3).

**Table 5:** Trabecular-iris space area at 750 μm (TISA) changes

Variable	Preoperative	First follow-up (1 month)	Second follow-up (3 months)	Third follow-up (6 months)	*P*-value
TISAS (mm^2^)	0.39 ± 0.04	0.16 ± 0.03	0.16 ± 0.03	0.16 ± 0.03	*P*1: < 0.001 *P*2: < 0.001 *P*3: < 0.001 *P*4: 0.935 *P*5: 0.388 *P*6: 0.057
TISAI (mm^2^)	0.41 ± 0.04	0.22 ± 0.04	0.23 ± 0.03	0.23 ± 0.03	*P*1: < 0.001 *P*2: < 0.001 *P*3: < 0.001 *P*4: 0.056 *P*5: 0.123 *P*6: 0.066
TISAN (mm^2^)	0.36 ± 0.01	0.23 ± 0.06	0.22 ± 0.06	0.22 ± 0.06	*P*1: < 0.001 *P*2: < 0.001 *P*3: < 0.001 *P*4: 0.070 *P*5: 0.089 *P*6: 0.695
TISAT (mm^2^)	0.37 ± 0.03	0.21 ± 0.06	0.21 ± 0.06	0.21 ± 0.06	*P*1: < 0.001 *P*2: < 0.001 *P*3: < 0.001 *P*4: 0.073 *P*5: 0.059 *P*6: 0.067

*P*1, pre versus first; *P*2, pre versus second; *P*3, pre versus third; *P*4, first versus second; *P*5, first versus third; *P*6, second versus third

##### The vault changes

The vault was 495.7 ± 68.5 µm at the first follow-up (1 month) and decreased to 487 ± 66.9 µm after 6 months of follow-up with no significant change (Table 6 and Fig. 1).

**Table 6:** The vault changes

Variable	First follow- up (1 month)	Second follow-up (3 months)	Third follow-up (6 months)	*P*-value
Vault (um)	495.7 ± 68.5	489.9 ± 67.7	487 ± 66.9	*P*1: 0.079 *P*2: 0.058 *P*3: 0.066

*P*1, first versus second; *P*2, first versus third; *P*3, second versus third

## Discussion

PIOLs are widely used for correction of some cases of refractive errors which are unsuitable for LASIK. The Visian Implantable Collamer lens (ICL-Staar Surgical AG, Nidau, Switzerland) and Implantable Phakic Contact Lens (IPCL, Caregroup Sight Solutions, India) are the most commonly used posterior chamber PIOLS. The safety and efficacy of ICL have been demonstrated for a long time, but the economic burden limits its use in some areas such as developing countries [7–9].

IPCL is a good less expensive alternative for the correction of errors of refractions up to − 30 D while the ICL can only correct up to − 18 D. The initial design of IPCL was V1 which required a PI, while the more recent V2.0 design has a central hole with reduced risk of pigment dispersion and pupillary block glaucoma and less incidence of cataract formation due to maintained aqueous current between the anterior capsule of lens and posterior surface of IPCL. [5].

The most common postoperative complications of pIOLs are cataract and glaucoma [10], and thus, an accurate IPCL size is very important and requires a proper preoperative accurate assessment of ACD and WTW diameter. [5] Many studies have shown that it is possible to accurately calculate the ICL size to ensure the optimum postoperative vault height (distance between ICL and crystalline lens). [11, 12] To prevent postoperative cataracts and glaucoma, the ideal size is very critical to detect the vault and angle of the AC.

To the best of our knowledge, this is the first study to report the AC angle changes after IPCL using AS-OCT. Angle evaluation is very important in the follow-up of patients with IPCL. Several imaging modalities can be used for this purpose such as Scheimpflug imaging, AS-OCT and ultrasound biomicroscopy (UBM). Our study showed that ACA, AOD and TISA decreased significantly after 1 month of IPCL implantation and remained stable during 6 months of follow-up with no significant change between 1, 3 and 6 months postoperatively. This indicates significant AC angle narrowing after IPCL implantation that remains stable thereafter.

The vault was measured manually as the distance between the posterior surface of the IPCL and anterior surface of the crystalline lens. Evaluation of the vault is very important as a lower vault has a major risk of cataract development and higher vault is a major risk for glaucoma. A safe vault value was considered to be 250–750 µm, and the lens should be explanted if the vault is close to 1000 µm. [13] In our study, the vault was 495.7 ± 68.5 µm at the first follow-up (1 month) and remained at a similar value after 3 and 6 months. This comes in agreement with previous studies such as Sachdev and Ramamurthy who implanted IPCL V1 and Bianchi et al. [5, 14] who implanted the V2.0 design.

IOP monitoring showed a slight increase at 1 month postoperatively which might be caused by retained viscoelastic, postoperative inflammation and/or topical steroid use. IOP then returned to near preoperative values for the rest of the follow-up period. This was concomitant with other studies such as Bianchi et al. [14, 15]. AS-OCT was used for angle evaluation with ICL as in the study by Singh et al. who implanted ICL in 32 eyes and found stable angle narrowing over 3 months of follow-up as well as Gargallo-Martinez who studied the ICL vault using AS-OCT.

A study conducted by Wan et al. [16, 17] on 82 myopic eyes implanted with Visian ICL used 3 instruments: AS-OCT, Oculus Pentacam and UBM for assessment of ACD and central vault for 3 months. They demonstrated that AS-OCT measurements were higher while Pentacam measurements were lower than UBM measurements.[18].

Our study has some limitations, such as the limited follow-up time, the small sample size and the absence of angle assessment by other tools such as UBM or Pentacam.

## Conclusion

In conclusion, IPCL V2.0 is a safe approach for correction of refractive errors with no need for PI provided that lens size is accurate. Furthermore, AS-OCT is a safe non-contact method for AC angle and vault evaluation after IPCL implantation.

## Data Availability

Available upon request from the corresponding author.
